# An investigation of gecko attachment on wet and rough substrates leads to the application of surface roughness power spectral density analysis

**DOI:** 10.1038/s41598-022-15698-2

**Published:** 2022-07-07

**Authors:** Amanda M. Palecek, Austin M. Garner, Mena R. Klittich, Alyssa Y. Stark, Jacob D. Scherger, Craig Bernard, Peter H. Niewiarowski, Ali Dhinojwala

**Affiliations:** 1 Gecko Adhesion Research Group, The University of Akron, Akron, OH USA; 2 Integrated Bioscience Program, The University of Akron, Akron, OH USA; 3 Department of Biology, The University of Akron, Akron, OH USA; 4 Department of Polymer Science, The University of Akron, Akron, OH USA; 5 Present Address: Department of Biological Sciences, Clemson University, Clemson, SC USA; 6 Present Address: Department of Biology, Villanova University, Villanova, PA USA; 7 Present Address: Avery Dennison, Oegstgeest, The Netherlands

**Keywords:** Atomic force microscopy, Biomechanics, Herpetology

## Abstract

The roughness and wettability of surfaces exploited by free-ranging geckos can be highly variable and attachment to these substrates is context dependent (e.g., presence or absence of surface water). Although previous studies focus on the effect of these variables on attachment independently, geckos encounter a variety of conditions in their natural environment simultaneously. Here, we measured maximum shear load of geckos in air and when their toes were submerged underwater on substrates that varied in both surface roughness and wettability. Gecko attachment was greater in water than in air on smooth and rough hydrophobic substrates, and attachment to rough hydrophilic substrates did not differ when tested in air or water. Attachment varied considerably with surface roughness and characterization revealed that routine measurements of root mean square height can misrepresent the complexity of roughness, especially when measured with single instruments. We used surface roughness power spectra to characterize substrate surface roughness and examined the relationship between gecko attachment performance across the power spectra. This comparison suggests that roughness wavelengths less than 70 nm predominantly dictate gecko attachment. This study highlights the complexity of attachment in natural conditions and the need for comprehensive surface characterization when studying biological adhesive system performance.

## Introduction

Geckos can adhere to a variety of substrates, from rough tree bark and rock to smooth glass^1–4^. To adhere, geckos use expanded scales (scansors) on the subdigital surface of their digits which are covered with hair-like fibrils (setae) composed of corneous beta-proteins and lipids^5–7^. The setae of Tokay geckos (*Gekko gecko*) are ca. 2–4 μm in diameter and ca. 11–115 μm in length, depending on the location on the subdigital pad^8^. Tokay gecko setae branch and terminate into plate-like spatulae, which are ca. 100–200 nm wide^9–15^. As a gecko engages its subdigital pads, spatulae are loaded and sheared into alignment^1^. The intimate contact provided by the millions of spatulae carried on the subdigital pads of geckos allows them to use intermolecular forces (primarily van der Waals interactions, but research also suggests the presence of capillary and acid–base interactions depending on the substrate) to generate adhesive forces well in excess of that required to resist sliding on clean, smooth substrates with the toes properly oriented (e.g., > 40 N maximum shear load for 100 g gecko^16–21^).

The natural substrates that free-ranging geckos encounter are complex and their surfaces likely vary in wettability, surface roughness, hardness, and the presence of contaminants (e.g., dirt or water), among others^22,23^. Many of these conditions can significantly reduce or completely inhibit gecko adhesion by preventing the adhesive contact required for intermolecular forces to function. For example, surface water can reduce gecko adhesion on hydrophilic substrates because water infiltrates the contact interface between the subdigital pad and the surface, disrupting the van der Waals adhesion^24,25^. If the substrate is hydrophobic, however, geckos can maintain adhesion while underwater by inducing dry contact between the substrate and their superhydrophobic adhesive subdigital pads (i.e., van der Waals adhesion is maintained). On surfaces like polytetrafluoroethylene, in which fluorination results in substrate hydrophobicity, adhesion is higher underwater than in air^25,26^. Whole-animal experimental data matches predictions from a classical thermodynamic model developed using the Young-Dupré equation^25^ and shows that surface wettability and surface water (i.e., the presence or absence) strongly influence gecko attachment performance.

In contrast to surface water and wetting (although see Stark et al.^27^), the influence of contact area-reducing surface roughness on gecko adhesive performance has received much attention^9,22,28–33^, yet the hierarchical nature of the gecko adhesive system has made it difficult to predict how various roughness length scales influence adhesion. When a uniform material (e.g., a hard solid) contacts a rough surface, the smallest roughness length scales impact adhesion^34–36^. However, previous work suggests that surface roughness at the length scale of each of the hierarchical levels of the gecko adhesive system (spatulae, setae, scansors) results in the reduction of fibrillar adhesion^29,33,37^. Recent work on rough substrates has suggested more general, but contradictory, theories. Naylor and Higham^22^ suggest that gecko adhesion declines linearly with surface roughness, while Pillai et al.^32^ indicate that gecko adhesion on rough substrates is nonlinear. Therefore, it is not clear how gecko adhesion is impacted by the surface roughness of natural substrates that likely vary in roughness and surface chemistry over multiple length scales^9,30,31^.

While substrate wettability, the presence or absence of surface water, and surface roughness individually influence gecko adhesion^22,26,29,32,33^, they are rarely investigated jointly (although see Stark et al.^38^, which examined the joint effects of presence/absence of surface water and orientation of a rough surface pattern), and surfaces with varying RMS roughnesses often also vary in surface chemistry. In this study, we planned to test for the individual and interactive effects of three key variables in a gecko's environment on attachment: substrate wettability, surface water, and surface roughness. Each of the variables is known to have some effect on adhesive performance, but this study will be the first to examine the joint effects of these variables on performance. Understanding how these variables interact can shed light on how complex natural substrates, such as rough, moist, hydrophobic leaves, may affect performance in nature. To examine this, we measured whole-animal attachment on substrates that varied in wettability (hydrophobic and hydrophilic), surface treatment (i.e., presence or absence of water on the substrate surface), and surface roughness. Previous work investigating gecko adhesion on rough fluorinated substrates and patterned hydrophobic substrates demonstrated that adhesion was either unaffected or improved in the presence of water^26,38^. We expected the same to be true when geckos attached to wet and dry rough casts made of hydrophobic polyethylene, as hydrophobic substrates repel water and could allow the intimate contact needed for adhesion despite the presence of water^25^. Although gecko adhesion reduces on smooth hydrophilic substrates when wetted^24,25^, we predicted that attachment to a rough hydrophilic substrate would not differ when wetted. This prediction supports the hypothesis that surface roughness plays a more important role compared to substrate wettability or surface chemistry on fibrillar adhesive performance^39^. Furthermore, we predicted that gecko adhesion would be sensitive to surface roughness at the relevant length scales of the integrated system. For example, adhesion will be lowest at RMS roughness heights similar to whatever scale of the gecko hierarchical system is examined, such that roughness at the size of the spatulae should inhibit adhesion at the spatular level, or roughness at the size of the setae will inhibit adhesion at the setal level^29^.

## Results

### Substrate characterization

We used two grades of sandpaper to serve as the hydrophilic substrates (2500-grit and 2000-grit) and polyethylene (PE) cast from the sandpapers as the hydrophobic substrates. PE substrates were originally designed to be hydrophobic replicas of the sandpapers, but surface roughness characterization determined that our casting methodology did not create accurate replicas (Table 1). Therefore, our casting methodology produced 4 PE substrates that differed in roughness from each other and the sandpapers. We used the following naming conventions for our PE substrates: smooth, fine, intermediate, and coarse. Root mean square (RMS) height was calculated at two different scan sizes via atomic force microscopy (AFM; hereafter denoted as RMS_afm_; Table 1). RMS_afm_ of the rough substrates was between two (fine PE) and 29 (2500-grit sandpaper) times greater than the RMS_afm_ of the smooth PE substrate. The ranking of substrate roughness by RMS_afm_ varied depending on the scan size. The 2500-grit sandpaper had the greatest RMS_afm_ of the 10 µm × 10 μm scans, but was the third greatest in terms of RMS_afm_ for the 55 µm × 55 μm scans. The 2000-grit sandpaper, on the other hand, had the third greatest RMS_afm_ of the 10 µm × 10 μm scans, but had the greatest RMS_afm_ of the 55 µm × 55 μm scans. The ranking of substrate roughness by RMS_afm_ of the remaining substrates did not differ between scan sizes.

**Table 1:** Mean RMS height values characterized via atomic force microscopy (RMS_afm_) and optical profilometry (RMS_prof_), and water contact angle as a function of substrate.

Substrate	RMS_afm_ ($\upmu$m)		RMS_prof_ ($\upmu$m)	$\uptheta$_w_ ($^\circ$)
	10 $\upmu$m × 10 $\upmu$m Scan	55 $\upmu$m × 55 $\upmu$m Scan	260 $\upmu$m × 350 $\upmu$m Scan	
Smooth PE	0.03 ± 0.004	0.06 ± 0.008	0.04 ± 0.013	97 ± 4
Fine PE	0.06 ± 0.006	0.17 ± 0.016	0.10 ± 0.006	109 ± 2
Intermediate PE	0.25 ± 0.049	0.75 ± 0.128	0.28 ± 0.028	108 ± 3
Coarse PE	0.70 ± 0.065	1.44*	0.63 ± 0.105	122 ± 3
2500-grit Sandpaper	0.87 ± 0.023	1.17 ± 0.041	1.18 ± 0.037	56 ± 4
2000-grit Sandpaper	0.41 ± 0.034	1.55 ± 0.434	1.13 ± 0.028	Wet

Values are reported as mean ± 1 s.e.m. The 2000-grit sandpaper was immediately wetted by water, thus the water contact angle for this substrate is listed as wet. *Only one scan was able to be completed on this substrate. PE = polyethylene.

**Figure 1:**
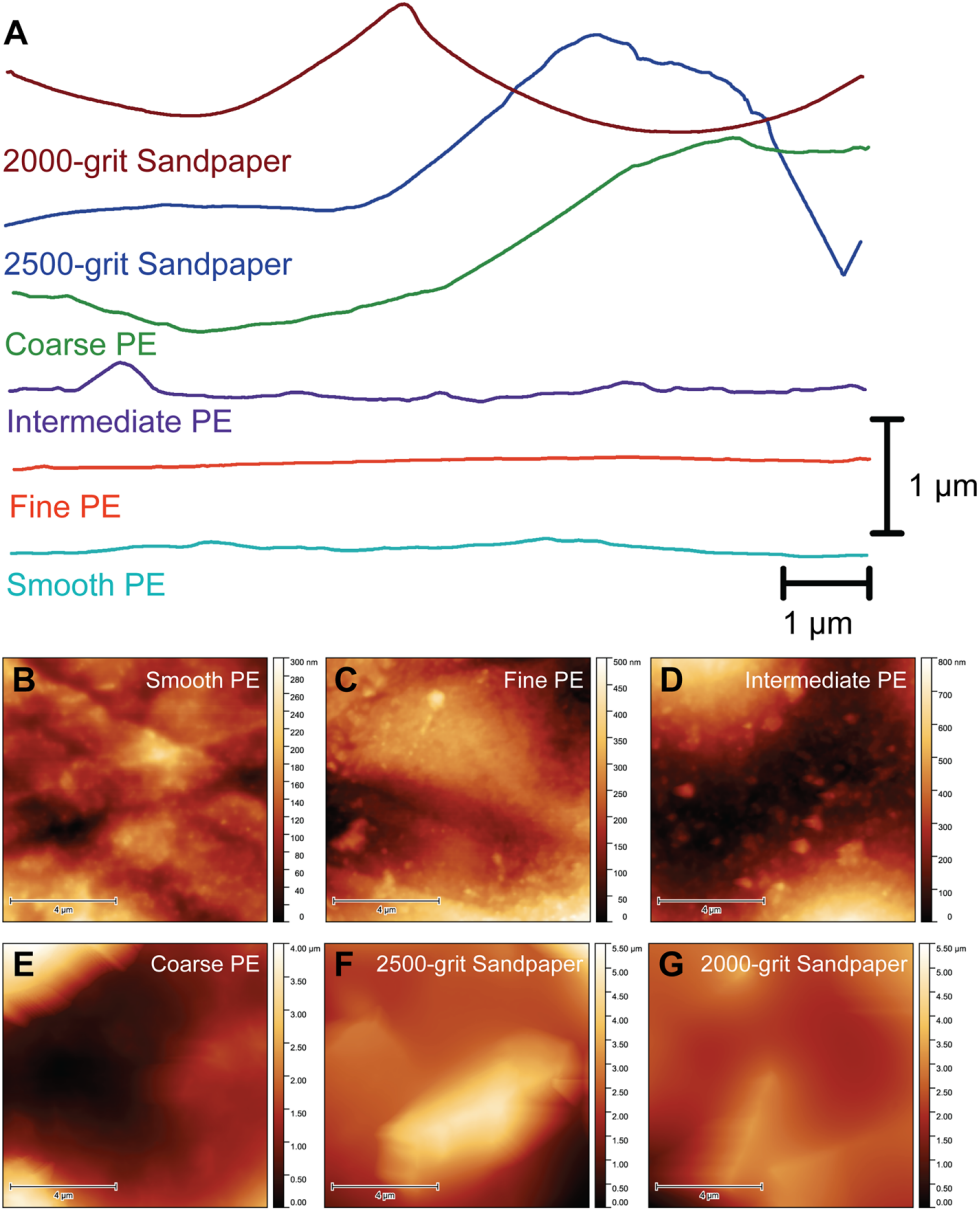
(**A**) Representative line scans of each substrate used in this experiment. Line scans of 10 μm in length were obtained along the x-axis of the representative 10 µm × 10 µm atomic force microscopy (AFM) scans shown in (**B**–**G**). Horizontal line scans were centred on the y-axis. (**B**–**G**) Representative 10 µm × 10 µm AFM scans of the substrates used in this experiment. PE = polyethylene.

We characterized substrates using optical profilometry to quantify surface roughness at larger length scales (i.e., larger than the 55 µm × 55 μm AFM scan size^40^). Three 260 μm × 350 μm scans were obtained per substrate and RMS height (hereafter denoted as RMS_prof_) was calculated for each scan. The ranking of substrate roughness based on RMS_prof_ was somewhat different than either RMS_afm_ scan size with the coarse PE having the third greatest RMS_prof_, the 2000-grit sandpaper having the second greatest RMS_prof_, and the 2500-grit sandpaper having the greatest RMS_prof_. RMS_prof_ of all substrates was almost always greater than the RMS_afm_ obtained from the 10 µm × 10 μm scans, but was almost always lower than the RMS_afm_ obtained from the 55 µm $\times$ 55 μm scans. Representative line scans and topography maps (obtained via 10 µm × 10 µm AFM scans) of each substrate are shown in Fig. 1.

Water contact angles (θ_w_) of all substrates were measured via goniometer (see "Methods" for details). θ_w_ of all the PE surfaces were well above 90°, indicating that these surfaces were hydrophobic (i.e., θ_w_ ≥ 90°). In contrast, the sandpaper surfaces had θ_w_ that were less than 90°, consistent with hydrophilic surfaces (Table 1). The 2000-grit sandpaper was immediately wetted by water droplets during θ_w_ measurements (θ_w_ is likely less than 10°; Table 1).

### Whole-gecko attachment

**Figure 2:**
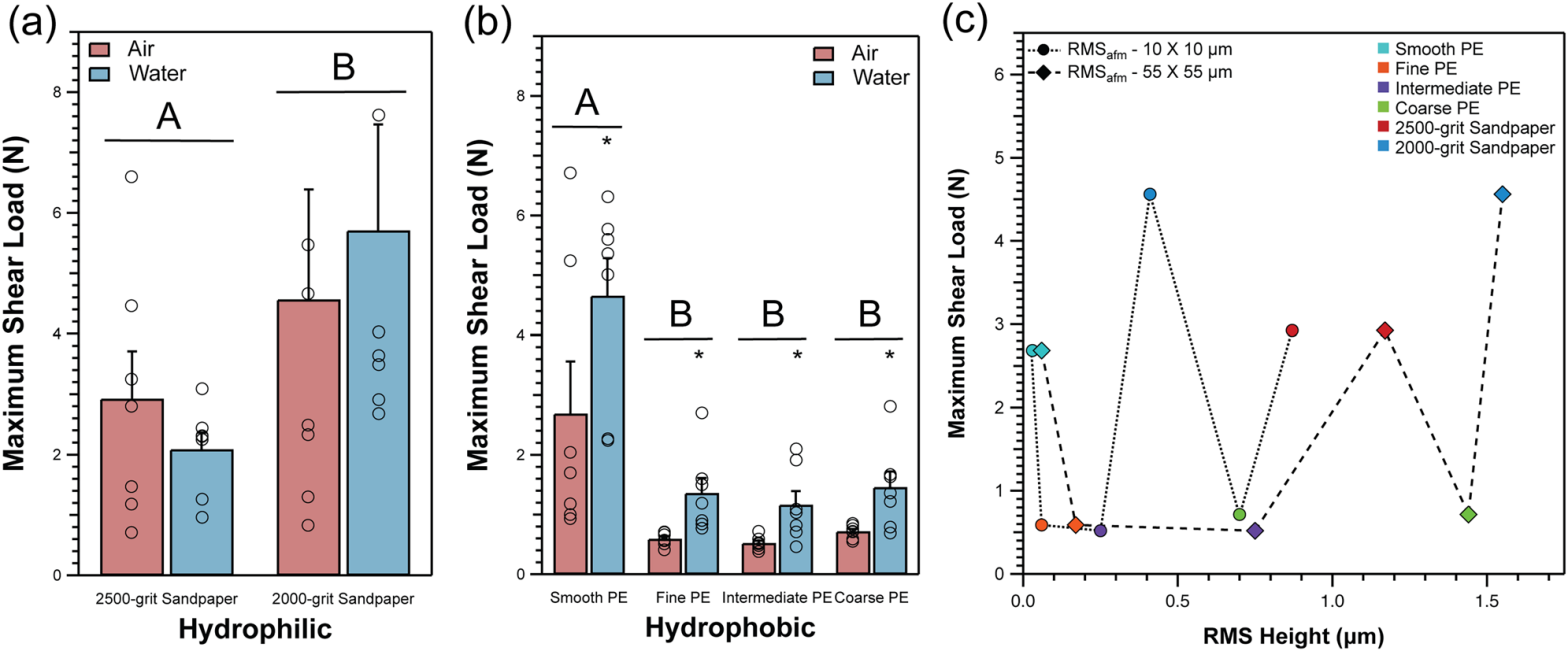
(**a**) Mean maximum shear load of live geckos tested on 2500-grit and 2000-grit hydrophilic sandpapers. (**b**) Mean maximum shear load of geckos tested on four hydrophobic polyethylene (PE) substrates that vary in surface roughness. Bars with different letters indicate statistically significant differences in adhesive performance as a function of substrate (i.e., bars with the same letter are not statistically different from one another). Stars represent statistically significant differences in adhesive performance as a function of surface water treatment (presence or absence; i.e., means without a star are not statistically different from one another). Open circles represent the raw data points of each individual gecko. Error bars are mean ± 1 s.e.m. (**c**) Mean maximum shear load of live geckos in air as a function of RMS height measured via AFM (RMS_afm_; at two different scan sizes). Lines merely connect individual points; they are not statistical fits.

Maximum shear load of 7 adult Tokay geckos (*Gekko gecko*) varied in response to surface roughness (i.e., 2500-grit and 2000-grit sandpaper and smooth, fine, intermediate, and coarse PE substrates), surface wettability (i.e., hydrophobic PE substrates and hydrophilic sandpapers), and surface treatment (i.e., presence and absence of surface water). Specifically, on hydrophilic sandpaper substrates, maximum shear load was marginally greater on the 2000-grit sandpaper than on the 2500-grit sandpaper (p = 0.0308; Fig. 2A, Table 2), however there was no effect of surface water on maximum shear load (p = 0.8183, see Table 2). On hydrophobic PE substrates, the presence of surface water yielded significantly higher maximum shear load than when surface water was absent (p < 0.0001). Surface roughness of PE substrates had a significant effect on maximum shear load (p < 0.0001), however this result was driven by only the smooth substrate. Specifically, maximum shear load on the smooth PE substrate was higher than all other PE substrates, but there was no significant difference between maximum shear load on the fine, intermediate, or rough PE substrates (Fig. 2B; Table 3).

**Table 2:** Statistical results of maximum shear load performance across all tested substrates and treatments.

Maximum shear load comparisons across substrates and treatments			
	df	Chi-squared	P value
**Sandpapers**			
Substrate	1	4.663	0.0308
Treatment	1	0.053	0.8183
**Polyethylene**			
Substrate	3	23.623	< 0.0001
Treatment	1	15.536	< 0.0001

**Table 3:** Tukey post-hoc test results from maximum shear load performance across polyethylene substrates.

	Smooth PE	Fine PE	Intermediate	Coarse PE
Smooth PE	–	P < 0.0001	P < 0.0001	P < 0.0001
Fine PE	P < 0.0001	–	P = 0.8857	P = 0.9311
Intermediate PE	P < 0.0001	P = 0.8857	–	P = 0.5492
Coarse PE	P < 0.0001	P = 0.9311	P = 0.5492	–

Figure 2C displays mean maximum shear load in air as a function of RMS height as measured by AFM and optical profilometry (Table 1). There is no obvious relationship between maximum shear load and RMS height. For example, the fine and coarse PE substrates vary in RMS_afm_ as obtained from the 55 µm × 55 μm scan by a factor of 8.5 (0.17 ± 0.016 μm vs. 1.44 μm), yet maximum shear load did not differ significantly between them (Fig. 2C).

### Surface roughness power spectra

**Figure 3:**
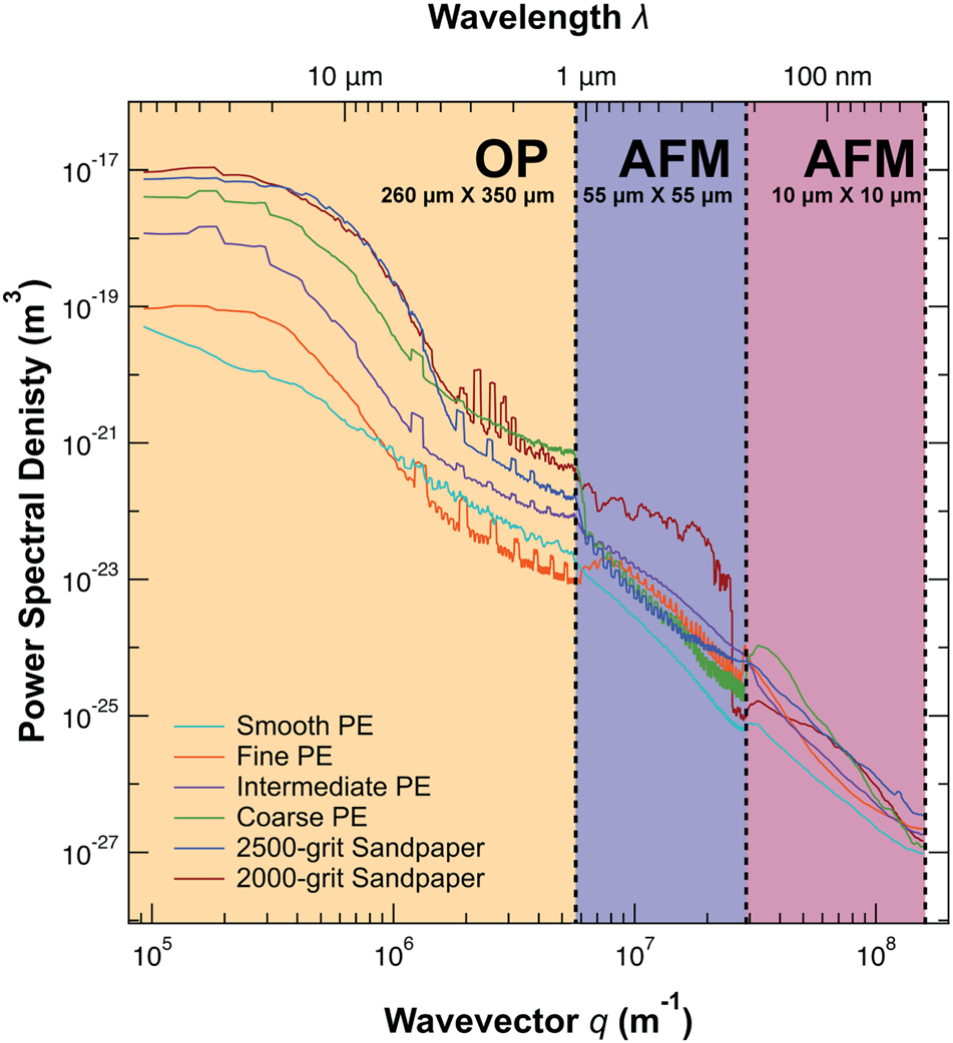
One dimensional surface roughness power spectral density (PSD) functions for each experimental substrate as a function of wavelength (λ) and wavevector q = 2π/λ. The PSDs illuminate qualities of the surfaces that were otherwise unknown via comparisons of RMS height. Regions of the figure with different fill colours indicate the primary range of wavelengths measured by each technique (OP = optical profilometry; AFM = atomic force microscopy). Note that the dashed vertical lines are not hard cut-offs; the data from each technique and/or scan size overlaps with at least one other technique and/or scan size, yet most data from each technique/scan size lies within the indicated regions.

Recent surface metrology work has warned that critical information about surfaces and their properties can be masked when roughness is simplified to RMS height, particularly when surface topography is measured at limited length scales^40,41^. Surface roughness power spectra or power spectral density (PSD) functions have been suggested as a powerful analytical tool to combat the limitations of RMS height^31,41–43^. Power spectral density (PSD) is calculated as the Fourier transform of a surface’s height-height autocorrelation function (see "Methods"; Eq. 1) and represents the contributions of sine waves of wavelength λ (more commonly expressed as wavevector q = 2π/λ) that compose a surface’s profile. Therefore, we calculated the PSD functions of each of our substrates from topography data obtained via optical profilometry and atomic force microscopy (Fig. 3; see "Methods"). As expected, PSD generally declines with increasing wavevector q. The PSD functions, however, illuminate qualities of the surfaces that were otherwise unknown. Most notably, the ranking of substrate roughness by PSD changes based on wavelength. For example, what we qualitatively and quantitatively (via RMS height) considered to be the least rough substrate (smooth PE) was as rough or rougher than the fine PE substrate at wavelengths between 1–6 μm. In fact, all six PSD functions intersect with at least one other substrate’s PSD function at some wavevector.

## Discussion

We investigated gecko adhesive performance on substrates that varied in wettability, the presence or absence of surface water (wet versus dry conditions), and surface roughness to examine how multiple environmentally relevant factors influence gecko attachment. Overall, we found that gecko maximum shear load was higher underwater than in air on hydrophobic substrates, regardless of surface roughness. We also found that attachment did not differ significantly as a function of surface water on rough hydrophilic substrates. When considering the impact of surface roughness on gecko adhesion, we consulted recent surface metrology work that provided critical insight into the effective measurement of surface roughness, leading us to employ new techniques and information not generally applied to gecko adhesion. In the following sections, we detail our findings and discuss a new hypothesis that describes how surface roughness influences gecko adhesion.

### Effects of substrate wettability and the presence/absence of water

On smooth hydrophilic glass, the addition of surface water is detrimental to maximum shear load^25^. However, the results of this study show that attachment to rough hydrophilic substrates is not influenced by surface water. This implies that surface roughness does not negatively affect adhesive performance on submerged, rough, hydrophilic substrates. Experimental and theoretical work has suggested that capillary-like interactions, microbubbles, or other mechanisms caused by increased surface roughness could enhance adhesion to wet hydrophilic substrates^44,45^. On smooth hydrophilic substrates, the presence of water reduces adhesion when the toe pads are fully wetted^24^. Our results suggest that the loss of dry contact reduces performance on hydrophilic substrates, and roughness asperities may allow pockets of dry contact between toe pad and substrate, though these air pockets would be too small to detect with the naked eye. In contrast, gecko attachment was significantly higher on rough and smooth hydrophobic substrates (PE) when submerged under water. The improvement of adhesive performance underwater on hydrophobic substrates has been described elsewhere on smooth and rough substrates, and is likely driven by (1) the ability of superhydrophobic gecko toe pads to expel water at the contact interface and (2) the increase in surface energy between the hydrophobic surface and the water^26,38^. On rough substrates however, surface asperities may hold pools of water between the gecko foot and substrate. Future studies should focus on determining the presence or absence of thin water layers or air bubbles between adhesive gecko setae and smooth and rough submerged substrates that vary in wettability. On both substrate types, the effect of surface water on attachment (i.e., no effect on hydrophilic sandpaper and improved attachment on hydrophobic PE) remained consistent regardless of the magnitude of surface roughness. Our results show that gecko adhesion can be maintained or even improved on the selected wet, rough hydrophilic and hydrophobic substrates, suggesting that gecko attachment to most natural substrates is robust, even when wet until the setae are fully wetted.

### Gecko attachment and surface roughness

Whole-gecko attachment varied considerably as a function of surface roughness. Comparing the two sandpaper substrates, maximum shear load was higher on the sandpaper with larger particles (2000-grit sandpaper) than the sandpaper with smaller particle sizes (2500-grit sandpaper). On the PE substrates, maximum shear load on the smooth PE was highest, and significantly reduced on all other PE substrates. This finding is supported by previous data using hydrophobic patterned substrates, where geckos also experienced a drop in adhesive performance compared to smooth/unpatterned substrates^38^. Previous work also suggests that gecko adhesion decreases when particular statistical parameters of surface roughness (e.g., RMS height) correspond to length scales of features comprising the hierarchical system (i.e., spatulae, setae, scansors)^29,33,46,47^. For example, Huber et al.^29^ measured the adhesion of gecko spatulae and live geckos, finding that adhesion is reduced when RMS height values are between 100–300 nm, corresponding to the approximate size of spatulae. Our findings, however, do not support those of Huber et al.^29^. If we use our calculations of RMS height from the 10 µm × 10 µm AFM scans (as Huber et al.^29^ did), gecko maximum shear load is indeed low on the fine PE and intermediate PE substrate which have RMS heights of ~ 60 nm and 250 nm, respectively. The coarse PE substrate, however, has an RMS height of ~ 700 nm and, based on the findings of Huber et al.^29^, we should have observed a significant increase in maximum shear load compared to the fine and intermediate PE substrates. Instead, maximum shear load on the coarse PE substrate did not differ significantly between the other three PE substrates suggesting that there is no simple relationship between maximum shear load and RMS height. Of course, there are likely many differences between our substrates and those examined in Huber et al. (e.g., lateral sizes of asperities), but such qualities are rarely quantified or discussed in gecko attachment literature and instead RMS height is used. Indeed, recent advancements in surface metrology have revealed that individual parameters such as RMS may not capture all the information about surface topography that drives variation in adhesion^40,41^.

#### Limitations of RMS height

RMS height is highly dependent on scan size and instrument resolution. Smaller scan sizes generally result in lower RMS height because lower spatial wavelengths are included in the data^48^. Techniques such as atomic force microscopy are capable of resolving smaller scale variation in roughness and thus RMS height is often lower. When switching techniques, there is also an expected drop in RMS (as seen between AFM and Optical Profilometry). This is a result of the optical profilometry being limited in its x–y–z-resolution relative to AFM, and information on the smaller wavelengths of roughness is lost. Additionally, several contemporaneous studies have highlighted the long-known notion that two surfaces with very different profiles can have identical RMS height values^40,41,49^. Characterization of our experimental substrates using two different techniques and multiple scan sizes highlights the dependence of RMS height on scan size (Table 1). For example, the RMS_afm_ of the 2500-grit and 2000-grit sandpapers increases by 1.3 and 3.8 times, respectively, when the scan size increases from 10 µm × 10 μm to 55 µm × 55 μm. Furthermore, when maximum shear load is plotted against RMS height obtained from each of the scan sizes and instruments, the overall shape of the trends differs substantially (i.e., RMS heights vary considerably depending on the scan size, complicating the substrate roughness to adhesive performance relationship; Fig. 2C). Thus, conclusions of the effect of surface roughness on macroscopic phenomena (e.g., adhesion, friction, wettability) using RMS height could be misleading if the methods of characterization are not taken into consideration^40,41^.

#### Surface roughness power spectra

Surface roughness power spectral densities (PSDs) are relatively unaffected by variables like scan size and instrument resolution, thus they may offer additional insight not provided by RMS height^40^. To be effective, PSDs should be generated over all relevant surface roughness wavelengths, and there are two main approaches to accomplish this: (1) generate PSDs for surface topographies measured via AFM and extrapolate to other relevant wavelengths, especially to those in the sub-nanometre range; (2) measure surface topography with multiple measurement techniques and stitch the PSD functions together^40^. Although Gujrati et al.^40^ found that the former method generates similar results to the latter method, they cautioned that the generalisability of their results is unknown and instead advised researchers to employ the latter method. Thus, we used this approach to generate PSDs that represented surface roughness over four orders of magnitude.

**Figure 4:**
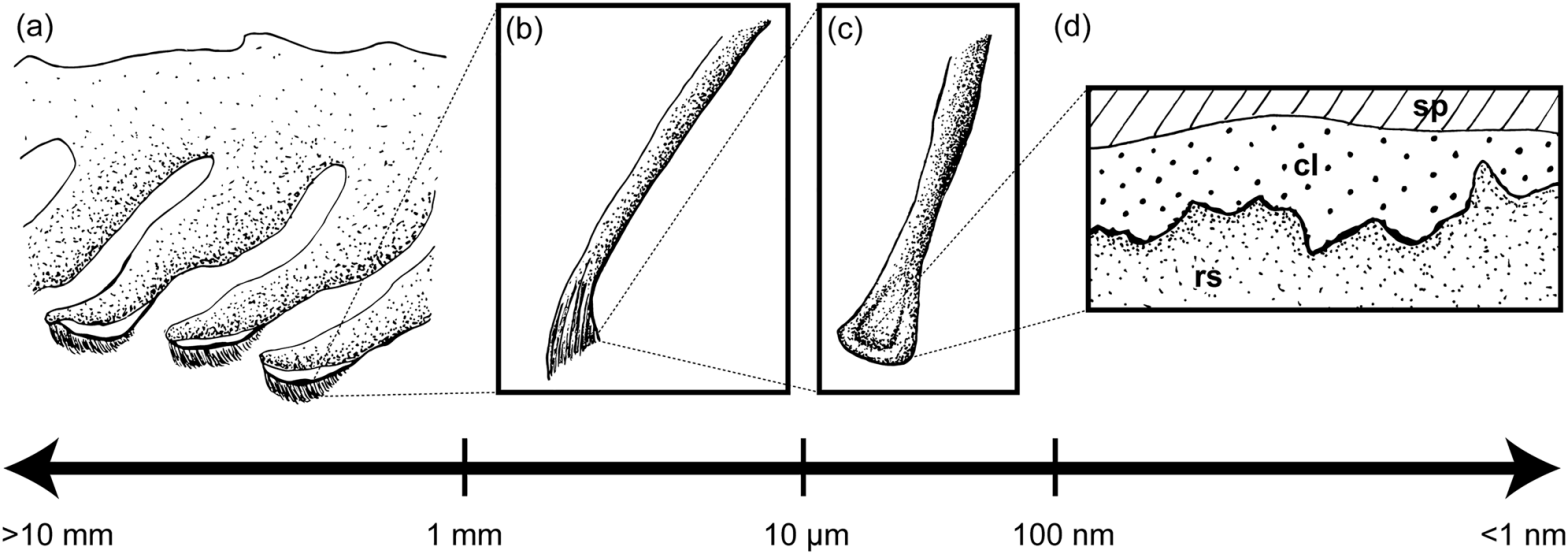
Illustration of which levels of the gecko adhesive hierarchy deform to wavelengths (λ) of roughness from the millimetre to nanometre scale as described by Persson ^28^. (**a**) The skin and underlying digital anatomy conform to surface roughness wavelengths of 1 mm and longer. (**b**) Gecko setae conform to roughness wavelengths between 10 μm and 1 mm. (**c**) Spatulae conform to surface roughness between wavelengths of 100 nm and 10 μm. (**d**) Persson^28^ proposed a thin, conformal layer on gecko spatulae that conforms to surface roughness wavelengths of 100 nm and shorter; sp = spatula; cl = conformal layer; rs = rough surface. Figure not drawn to scale.

**Figure 5:**
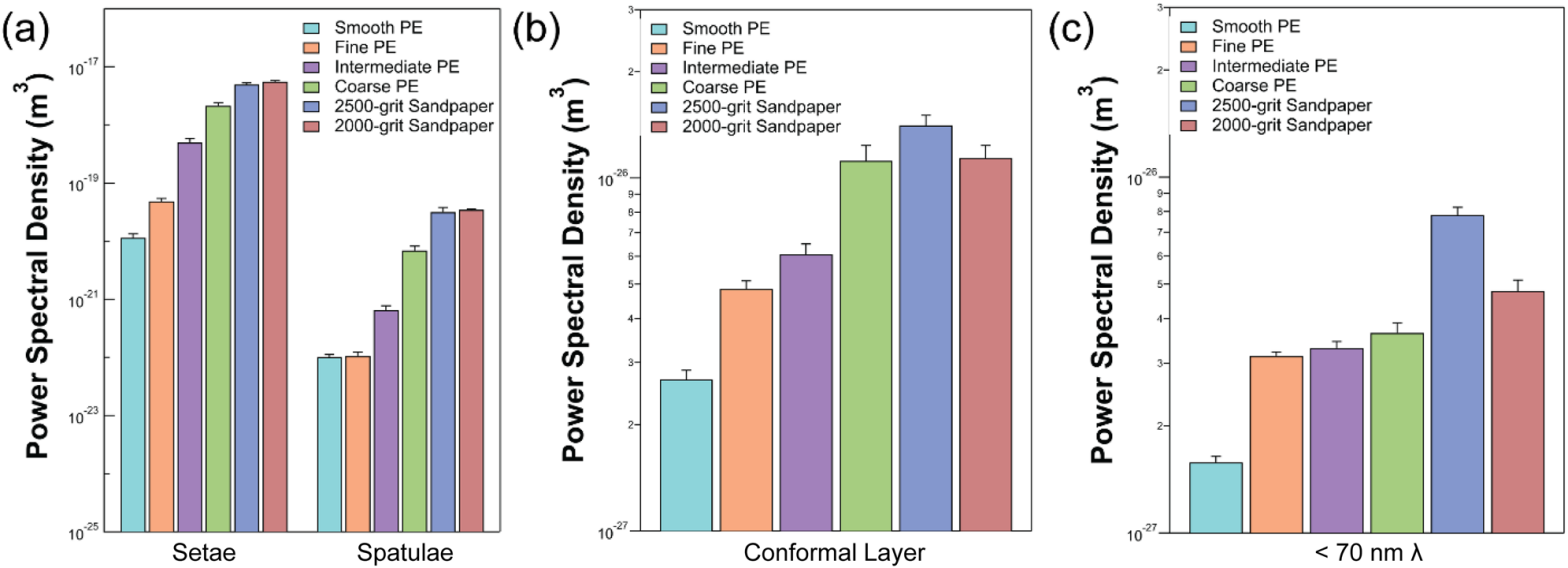
(**a**,**b**) Power spectral density (PSD) averaged over wavelengths of surface roughness described by Persson^28^. Setae and spatulae length scales (defined in Fig. 4) are displayed in (**a**), while the conformal layer length scale is displayed in (**b**). Note that roughness at the length scales of the skin/underlying digital anatomy (as defined by Persson^28^) was not present in the substrates. Variation in mean PSD between substrates does not correspond to our live gecko attachment experiments at any of the levels described by Persson^28^. (**c**) Mean PSD over wavelengths less than 70 nm. Variation in mean PSD is consistent with our maximum shear load data obtained with live geckos.

Persson^28^ qualitatively described which levels of hierarchy present in the gecko adhesive system (skin, setae, spatulae, thin conformal layer) should be responsible for deforming to surface roughness at particular wavelengths, thereby governing contact and adhesion (Fig. 4). By averaging our PSDs across the ranges of length scales described by Persson^28^, we can qualitatively assess whether trends in attachment correspond to the trends in roughness within those particular length scales (Fig. 5). Based on the assumption that increased surface roughness (i.e., greater power) reduces adhesion, we might expect the three ‘rough’ PE surfaces (fine, intermediate, coarse) to be similar in PSD and the 2500-grit sandpaper to be greater in PSD than the 2000-grit sandpaper. In contrast to this, the three ‘rough’ PE substrates differ substantially in PSD and the PSD of the two sandpapers are nearly identical at the three length scales examined (Fig. 5). Therefore, it is not clear how we can relate the quantitative information obtained in this experiment (whole animal attachment and surface roughness power spectra) to the qualitative approximations proposed by Persson^28^.

The calculation of surface roughness power spectral densities of our experimental substrates has generated a new qualitative hypothesis for future study. We scanned the PSD functions of our substrates to determine if there was a particular range of wavelengths for which the ranking of surface roughness by PSD matched the observed trends in gecko attachment. Specifically, we found that if PSDs are averaged over roughness wavelengths < 70 nm, the trends in roughness correspond to our attachment results. Figure 5C demonstrates that the three ‘rough’ PE surfaces are all similar in roughness (i.e., PSD) while the smooth PE surface is considerably smoother than the ‘rough’ PE surfaces. Furthermore, the 2500-grit sandpaper surface is much rougher than that of the 2000-grit sandpaper surface. These findings suggest that gecko adhesion is strongly sensitive to surface roughness at wavelengths < 70 nm, though more research is necessary to empirically test this. Methodologically, our equipment is capable of characterizing our experimental substrates down to the nanometre scale, however other work has utilized tools such as transmission electron microscopy to visualize surface roughness at the angstrom level^40^. As such, we are unable to determine whether surface roughness at length scales smaller than those measured with atomic force microscopy vary similarly to our attachment data. Future studies should consider utilizing well-characterized substrates with surface roughness that varies in wavelengths near or < 70 nm to test our hypothesis and that there are no interactive effects of maximum shear load performance with longer wavelengths of surface roughness.

## Conclusion

The interaction between a biological adhesive system and a substrate is dependent on substrate characteristics (e.g., surface roughness, wettability). The results of our study show that substrate roughness and wettability independently and jointly influence gecko adhesive performance on wet and dry substrates. Unintentionally, we found that the influence of surface roughness on gecko attachment is highly dependent on characterization methodology, a finding that is supported by recent surface metrology work. Characterization of our substrates across multiple length scales confirmed that RMS height is highly sensitive to variation in the scan size measured and instrument(s) used, thus rendering conclusions based on these values problematic if characterization methods are not taken into consideration. By measuring surface roughness over four orders of magnitude and calculating surface roughness power spectra, we discovered a range of surface roughness wavelengths (< 70 nm) that appear to qualitatively correlate with gecko attachment in our study and should be further investigated with well-characterized and highly controlled surfaces. Our study emphasises that the accurate characterization of a substrate is essential to understanding the interaction between biological adhesives and the substrate, which permits elucidation of the constraints imposed upon these systems. Developing theoretical models based on biological systems and specifying parameters for surface roughness characterization in gecko adhesion has direct implications to advances in evolutionary biomechanics, functional morphology, and bio-inspired design.

## Methods

### Surface preparation and characterization

To test the effect of surface wettability and roughness on gecko maximum shear load, we used commercially available hydrophilic sandpaper (9 × 11″ 3 M Wet/Dry sandpapers, Lee Valley Tools, Ontario Canada) which differed in approximate asperity size (either 2500-grit or 2000-grit). Surface chemistry was consistent (i.e., silicon carbide surfaces), but a smooth substrate with the same surface chemistry could not be obtained. Three additional substrates were formed from these sandpapers to attempt to create hydrophobic replicas as detailed below. An un-pressed polyethylene substrate was used as the smooth substrate.

**Figure 6:**
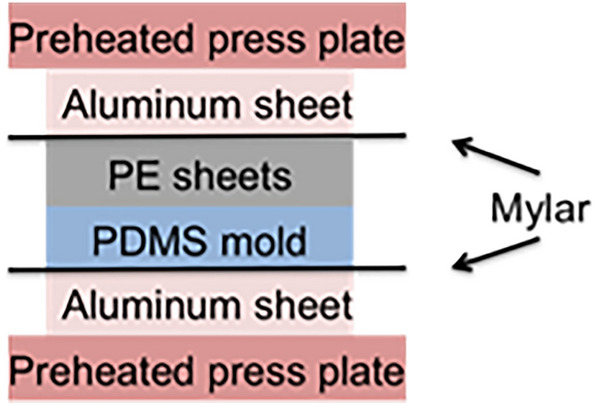
Components used in moulding assembly. Stainless steel plates supported the assembly, followed by an aluminium plate, mylar backing, the PDMS mould, and the PE sheets to be moulded.

Hydrophobic substrates with varying roughness were made using the sandpaper substrates as templates by a moulding process, followed by a compression pressing procedure. To form the negative mould, 80 g of polydimethylsiloxane (PDMS) was prepared, cast over the sandpaper, and cured at 40 $^\circ$C for 16 h. The sandpaper was peeled from the cured PDMS negative mould, and 2 sheets of polyethylene (PE) film were carefully rolled over the newly revealed surface. These were then placed between two preheated metal press plates (Fig. 6). A Drake hydraulic press was used to degas any remaining air between the PDMS and the PE by compressing the assembly under 4689 kPa, followed by a quick release of pressure. After degassing three times, the press was maintained at 110 °C, near the glass transition temperature for PE, for 30 min at < 234 kPa. The low pressure was used to minimize excessive mould compression while encouraging the infiltration of the PE into the cavities. The assembly was then removed from the press and allowed to air cool for one hour. The PE substrate was peeled from the PDMS mould and cured onto a glass substrate using a thin layer of PDMS. Previous studies revealed that macroscale substrate modulus had no effect on adhesion^50^. Substrate characterization (detailed below) revealed that the PE substrates were not adequate replicates of the sandpaper substrates. As such, four PE substrates with variation in macroscopic roughness (smooth, fine, intermediate, and coarse) were used for the experiment.

Surface roughness was characterized using two different methodologies^40^. First, the microroughness of each sample was determined using a Veeco Dimension Icon Atomic Force Microscope (Veeco; Plainview, NY, USA). All measurements were performed in tapping mode. Silicon tips (MikroMasch OÜ, Tallinn, Estonia) were 10 nm in average diameter, and had a drive frequency of 150 kHz, and a nominal spring constant of 5.4 N/m. Two scans (10 μm × 10 μm and 55 μm × 55 μm) per substrate per scan size were performed at each substrate location (except only one 55 µm × 55 μm scan for the rough PE substrate). By following a 10 μm scan with a 55 μm scan at the same location, it was confirmed that the tip was not damaging the polymer sample. Scan rate, setpoint, and gain were adjusted as necessary at each location to obtain the best image. NanoScope Analysis v1.2 software (Bruker Corporation, Billerica, MA, USA) was used to analyse the images obtained. Each image was first flattened to eliminate the effects of substrate orientation. RMS height was then calculated for each scan area using the software (Table 1). Substrate surface roughness was also characterized using an optical profilometer (Zygo NewView 7300; Zygo Corp., Middlefield, CT, USA). Using optical profilometry, three scans at three different locations on each substrate were performed at 20 × magnification. Average RMS height values were obtained from each substrate from the scans. In some cases, the height of small areas of the surfaces were unable to be resolved by the profilometer. In such cases, interpolation was used to fill-in the missing areas. Finally, water contact angle (θ_w_) of each substrate was measured using a Ramé-Hart Instruments Advanced Goniometer 500 F1 with DROPimage Advanced Software (Ramé-Hart Instruments Co., Succasunna, NJ, USA). At least 3 measurements were taken on each substrate, and the reported contact angles are the average ± 1 s.e.m. (Table 1).

### Whole animal attachment

Seven healthy mixed-sex adult Tokay geckos (*Gecko gekko*) were used to measure adhesive performance on substrates that varied in surface roughness, wettability, and presence or absence of surface water. Geckos weighed on average 96.6 ± 13.2 g. Geckos were housed individually in separate glass terrariums and exposed to a continuous cycle of 12 h of light followed by 12 h of darkness. They were fed a diet of cockroaches three times per week to ensure proper nutrition and a healthy weight. Cages were misted with water twice per day to keep experimental animals hydrated^18^. Before each trial, animals were acclimated to the experimental conditions for thirty minutes. An ambient temperature of 24.1 ± 0.2 $^\circ$C, humidity at 35 ± 5% RH, and water temperature (if applicable) of 22.8 ± 0.1 $^\circ$C was maintained during acclimation period and throughout experimental trials. All methods were performed in accordance with the relevant national guidelines and ethical regulations. The University of Akron IACUC protocol 07-4G approved all of the procedures used and the procedures were consistent with the guidelines provided by the Society for the Study of Amphibians and Reptiles (SSAR 2004). The study was carried out in compliance with the ARRIVE guidelines.

A force sensing apparatus, composed of a motor-driven force sensor (Shimpo FGV-10X; Shimpo Instruments, Glendale Heights, IL) that is displaced parallel to the substrate^18^, was positioned horizontally. The force sensor had a sampling frequency of 2000 Hz and a force resolution of 0.01 N. The motor was driven at a speed of 0.08 cm s^-1^. Geckos were placed on a substrate within a plastic bin and pulled with the force sensing apparatus via two harnesses (threaded through a window cut from the bin wall) attached at the pelvis (see Fig. S1). Geckos were induced to take a step on the substrate with all four feet before the experiment was started to ensure that the adhesive system was engaged. In water tests, the bin was filled with enough water (∼1 cm) to completely cover the foot and ankle on the substrate before placing the gecko into the bin. The force at which all four feet slipped on the substrate was measured and recorded as maximum shear load (N). All seven geckos were tested three times on each substrate underwater and in air. The highest force produced by individuals (n = 7) from each of the treatments was used in data analysis. Geckos had at least one day of rest between each set of experiments, totalling no more than three experimental measurements in a day. Geckos that were tested in water were not tested again without at least one day of rest to allow their feet to dry and prevent water from interfering with subsequent trials. Geckos were tested randomly on all substrates and treatment combinations and weighed after every experimental set.

### Statistical analysis

We used Kruskal–Wallis tests to test the effect of surface roughness, surface treatment (presence or absence of water) on gecko maximum shear load. Hydrophilic and hydrophobic cases (sandpapers and PE substrates, respectively) were separated and tested individually to examine if the effect of surface roughness and treatment on gecko maximum shear load differed between substrates (results are labelled in Fig. 2A,B, where different letters indicate significant differences). Post-hoc Tukey Honest Significant Differences (HSD) tests were used to determine differences in maximum shear load between treatment groups when appropriate. All statistical analyses were conducted in R (R Core Team, Vienna, Austria 2017) using the *lme4* package^51^.

### Surface roughness power spectra

Substrate topographies for both AFM and optical profilometry (OP) measurements were uploaded to a web-based tool (https://contact.engineering) to calculate power spectral density functions of each individual surface scan with each measurement technique (OP and AFM). The surface roughness power spectrum *C(q)* or power spectral density (PSD) can be generated via a Fourier transform of the surface’s height-height autocorrelation function. This is described in the following equation,

1 $$C\left(q\right)= \frac{1}{2\pi }\int \langle h\left(x\right)h(0)\rangle {e}^{-iqx}dx$$

where h(x) is the surface height deviation from the average surface height and <  > is the ensemble average^43^. One dimensional (1D) power spectra in the x scan direction was reported in this study. To obtain a single PSD function for each surface, the PSDs generated from multiple scans of the two different characterization methods (OP and AFM) were averaged and the resulting data sorted by wavevector q (q = 2π/wavelength λ). A running average with a window of 10 was used to combine the PSDs obtained via OP and AFM (Fig. 3).

### Ethical approval

All methods were performed in accordance with relevant guidelines and ethical regulations. The University of Akron IACUC protocol 07-4G approved all the procedures used and the procedures were consistent with the guidelines provided by the Society for the Study of Amphibians and Reptiles (SSAR 2004). The study was carried out in compliance with the ARRIVE guidelines.

## Data, code, materials

The datasets supporting this article have been uploaded as part of the supplementary material.

## Supplementary Information

Supplementary Information 1.

Supplementary Information 2.

Supplementary Information 3.

Supplementary Information 4.

Supplementary Information 5.
