# Supplementary figures and images for: An investigation of gecko attachment on wet and rough substrates leads to the application of surface roughness power spectral density analysis
Source: Sci Rep. 2022 Jul 7;12:11556. doi: 10.1038/s41598-022-15698-2 (PMC9262901; doi:10.1038/s41598-022-15698-2)

## Slide 1
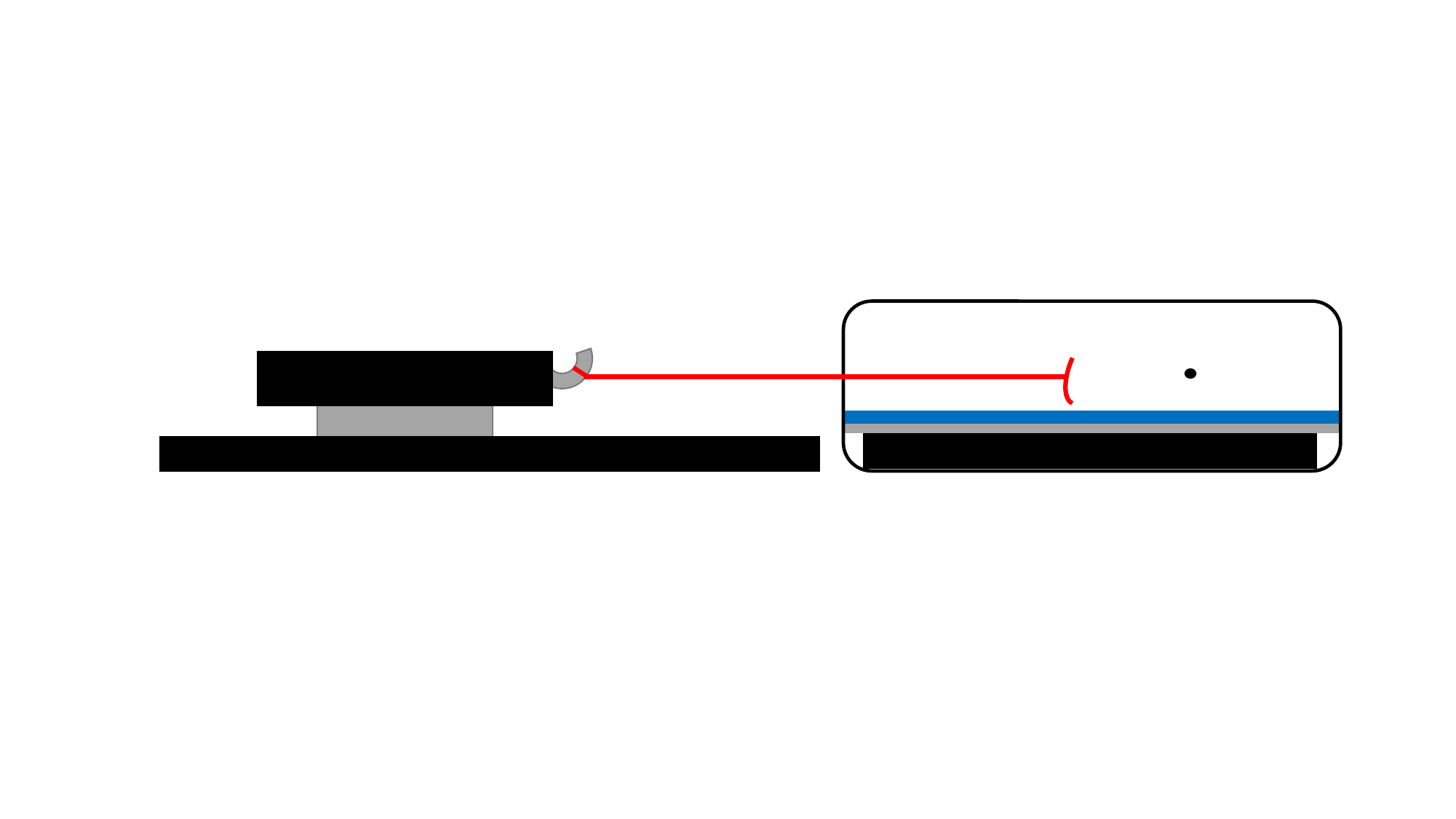

Supplement: Supplementary file 4 — Supplementary Information 4. [file 41598_2022_15698_MOESM4_ESM.pptx]
