# Supplementary material for: An investigation of gecko attachment on wet and rough substrates leads to the application of surface roughness power spectral density analysis
Source: Sci Rep. 2022 Jul 7;12:11556. doi: 10.1038/s41598-022-15698-2 (PMC9262901; doi:10.1038/s41598-022-15698-2)
Supplement: Supplementary file 5 — Supplementary Information 5. [file 41598_2022_15698_MOESM5_ESM.docx]

**An investigation of gecko attachment on wet and rough substrates leads to the application of surface roughness power spectral density analysis**

Amanda M. Palecek^1,3,5,^*, Austin M. Garner^1,2,3,6^, Mena R. Klittich^1,4,7^, Alyssa Y. Stark^1,2,3,6^, Jacob D. Scherger^4^, Craig Bernard^1,3^, Peter H. Niewiarowski^1,2,3^, and Ali Dhinojwala^1,2,4^

^1^Gecko Adhesion Research Group, The University of Akron, Akron, OH USA

^2^Integrated Bioscience Program, The University of Akron, Akron, OH USA

^3^Department of Biology, The University of Akron, Akron, OH USA

^4^Department of Polymer Science, The University of Akron, Akron, OH USA

^5^Present address: Department of Biological Sciences, Clemson University, Clemson, SC USA

^6^Present address: Department of Biology, Villanova University, Villanova, PA USA

^7^Present address: Avery Dennison, Oegstgeest, Netherlands

*Corresponding author. E-mail: [apalece@g.clemson.edu](mailto:apalece@g.clemson.edu)

**Supplementary Information Legends**

**Supplementary Figure 1. Schematic of live animal attachment data collection set up.**

**Supplementary Dataset 1. Gecko performance data on polyethylene substrates.** Adhesive performance data on hydrophobic polyethylene substrates used for statistical analyses.

**Supplementary Dataset 2. Gecko performance data on sandpaper substrates.** Adhesive performance data on hydrophilic sandpaper substrates used for statistical analyses.

**Supplementary Dataset 3. Power spectral densities across all tested substrates.**
